# Umbilical artery thrombosis risk factors and perinatal outcomes

**DOI:** 10.1186/s12884-024-06335-z

**Published:** 2024-02-14

**Authors:** Shuangjia Pan, Anjian Xu, Xinyue Lu, Baoyi Chen, Xianjun Chen, Ying Hua

**Affiliations:** 1 Department of Obstetrics and Gynecology, the Second Affiliated Hospital of Wenzhou Medical University, Wenzhou, 325027 China; 2 Department of Obstetrics and Gynecology, Taizhou Women and Childrens Hospital of Wenzhou Medical University, Taizhou, 325000 China

**Keywords:** Umbilical artery thrombosis, Isolated single umbilical artery, Single umbilical artery, Risk factors, Adverse perinatal outcomes

## Abstract

**Purpose:**

The purpose of this study was to investigate the risk factors for umbilical artery thrombosis (UAT) and the relationship between umbilical artery thrombosis and perinatal outcomes.

**Methods:**

This was a retrospective study that enrolled singleton pregnant women who were diagnosed with umbilical artery thrombosis. The control group recruited pregnant woman with three umbilical vessels or those with isolated single umbilical artery (iSUA) who were matched with umbilical artery thrombosis group. The risk factors and perinatal outcomes were compared between the groups.

**Results:**

Preconception BMI (OR [95%CI]: 1.212 [1.038–1.416]), abnormal umbilical cord insertion (OR [95%CI]: 16.695 [1.333-209.177]) and thrombophilia (OR [95%CI]: 15.840 [1.112-223.699]) were statistically significant risk factors for umbilical artery thrombosis. An elongated prothrombin time (OR [95%CI]: 2.069[1.091–3.924]) was strongly associated with the occurrence of UAT. The risks of cesarean delivery, preterm birth, fetal growth restriction, neonatal asphyxia, and intraamniotic infection were higher in pregnancies with UAT than in pregnancies with three umbilical vessels or isolated single umbilical artery (*P*<0.05). Additionally, the incidence of thrombophilia was higher in pregnant women with umbilical artery thrombosis than those with isolated single umbilical artery (*P* = 0.032). Abnormal umbilical cord insertion was also found to be associated with an elevated risk of iSUA (OR [95%CI]: 15.043[1.750-129.334]).

**Conclusions:**

Abnormal umbilical cord insertion was the risk factor for both umbilical artery thrombosis and isolated single umbilical artery. The pregnancies with umbilical artery thrombosis had a higher risk of the adverse perinatal outcomes.

**What is known**

- Umbilical artery thrombosis was a rare pregnancy complication, and due to its low incidence, the majority of relevant literature consisted of case reports.
- The diagnosis of Umbilical artery thrombosis could only be confirmed through postpartum umbilical cord pathological examination, as the ultrasound findings typically revealed the presence of only one umbilical artery during pregnancy.

**What is New**

- The risk factors for umbilical artery thrombosis included preconception BMI, abnormal umbilical cord insertion and thrombophilia.
- Abnormal umbilical cord insertion was the risk factor for both umbilical artery thrombosis and isolated single umbilical artery.
- Pregnant women with umbilical artery thrombosis were at an increased risk of adverse perinatal outcomes such as fetal growth restriction and preterm birth.

## Induction

The umbilical cord, serving as the link between mother and fetus, normally contains two arteries and one vein. In 0.5-2.0% of pregnancies [1, 2], one of the umbilical arteries may not develop or regresses progressively. The condition characterized by absence of one of the umbilical arteries is referred to as single umbilical artery (SUA). The most widely accepted underlying explanations for the causes of SUA include the primary agenesis, later atrophy of one umbilical artery or persistence of the original single allantoic artery of the body stalk [3]. Structural and chromosomal anomalies are considered to be closely related to the occurrence of SUA. It had been reported that approximately 33% of fetuses with SUA have additional structural anomalies, and 10% are affected with aneuploidy [4–7], implying that SUA severs as a soft marker for adverse pregnant outcomes. However, approximately 65% of cases with SUA are an isolated finding without fetal malformations or chromosomal abnormalities, which are referred to as isolated single umbilical artery (iSUA) [7].

Umbilical artery thrombosis (UAT) is a rare complication of pregnancy strongly associated with poor fetal and perinatal outcomes, such as intrauterine asphyxia, fetal growth restriction, and stillbirth [8–10]. UAT is considered to be a special type of single umbilical artery, since the ultrasound manifestation of UAT is the presence of only one umbilical artery during pregnancy and most pregnancies with UAT have no specific clinical symptoms or signs [8]. The diagnosis of UAT can only be confirmed by pathological examination of umbilical cord after delivery. Previous studies have shown an incidence of UAT between 0.025% and 0.045% [8, 9]. However, the scarcity of research on UAT is mainly attributed to the low incidence rate of UAT and the predominance of case report-based literature, which limits further investigation in the field.

Furthermore, many risk factors have been reported to be associated with the occurrence of SUA in pregnant women, including sex, multiple births, ethnicity, older maternal age, multiparity, and smoking, as well as the presence of maternal medical and pregnancy complications, maternal drug use [11–13]. However, it is worth noting that the existing literature lacks comprehensive reports that specifically investigate the precise risk factors of UAT.

Therefore, the purpose of this study was to investigate the risk factors for UAT, as well as the relationship between UAT and perinatal outcomes, which identify high-risk pregnancies with UAT earlier and provide evidence for perinatal health care of women with abnormal umbilical artery detected by ultrasound during pregnancy, thereby reducing the potential complications.

## Methods

### Study design

This was an observational retrospective study performed in four hospitals from January 1, 2016 to December 31, 2022. All pregnant women with umbilical artery thrombosis (UAT) identified during the study period were included as cases, while the pregnant women with isolated single umbilical artery (iSUA) or the pregnant women with three umbilical vessels during the same period were included as two distinct control group. All participants were between 20 and 45 years old and had singleton pregnancies conceived naturally. Exclusion criteria were women with twin pregnancies, fetal malformation, fetal chromosome aneuploidy, genetic abnormalities, assisted reproductive techniques or with incomplete medical histories and laboratory data. The Research Ethics Committees of the four participating hospitals approved this study.

### Diagnosis of UAT and iSUA

**Fig. 1:**
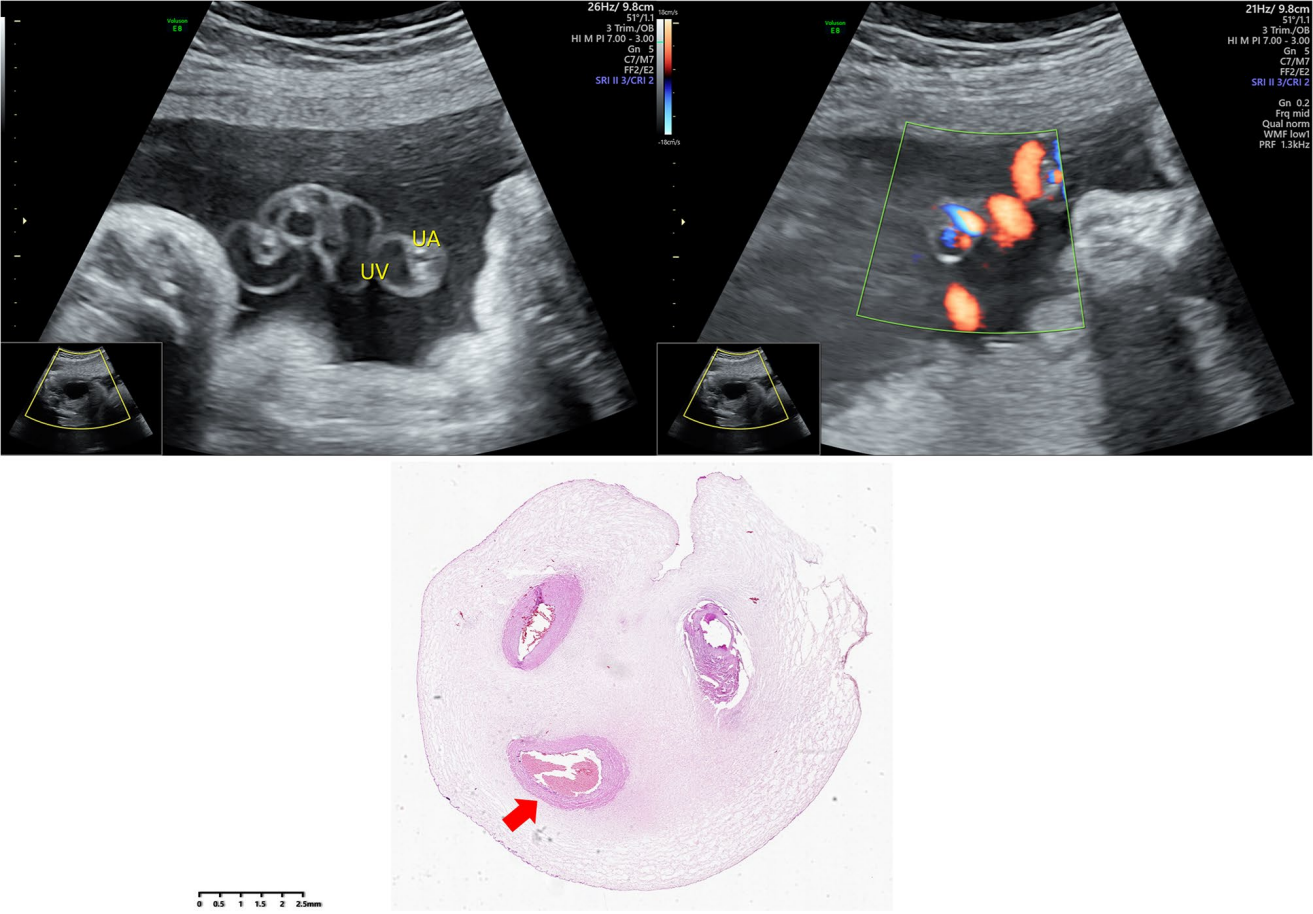
Ultrasound images and pathological staining of a single pregnant woman with umbilical artery thrombosis. UA: umbilical artery; UV: umbilical vein. *Arrows* indicate thrombosis of the umbilical artery

Umbilical artery thrombosis (UAT) was diagnosed when Ultrasonography revealed the presence of only one visible umbilical artery, whereas previous ultrasounds had indicated bilateral umbilical arteries. Additionally, the ultrasound identified a suspicious intraluminal low-echogenic substance within the umbilical artery, leading to the diagnosis of umbilical artery thrombosis. Subsequent pathological examination of the umbilical cord post-delivery confirmed the presence of umbilical artery thrombosis [14] (Fig. 1).

**Fig. 2:**
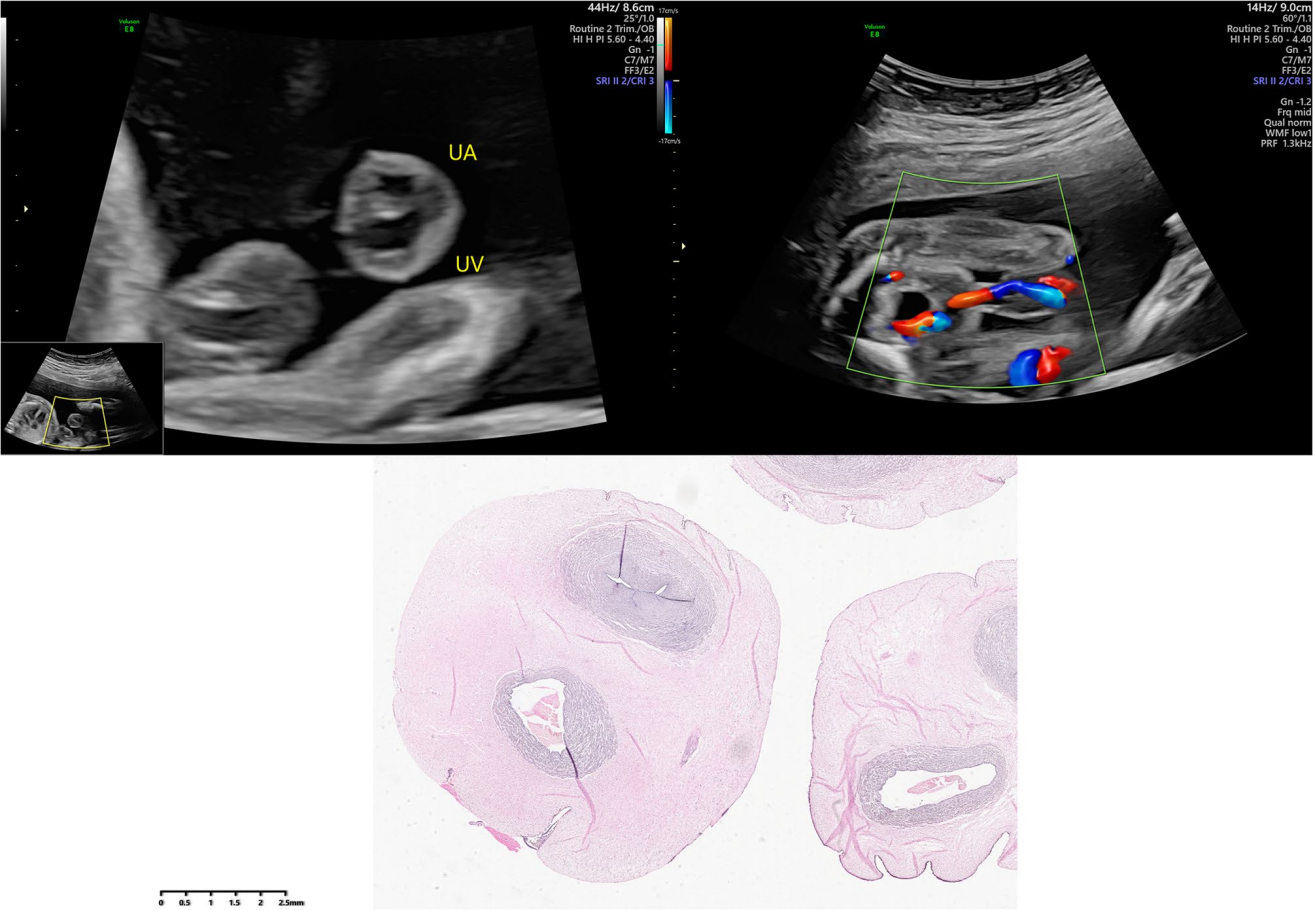
Ultrasound images and pathological staining of a single pregnant woman with isolated single umbilical artery. UA: umbilical artery; UV: umbilical vein

An isolated single umbilical artery (iSUA) was diagnosed at the time that during fetal anatomical scanning, the transverse section of the fetal pelvis revealed the presence of only a single umbilical artery encircling the fetal bladder, and color Doppler ultrasound confirmed the existence of a single umbilical artery without any fetal malformations or chromosomal abnormalities. Subsequent pathological examination of the umbilical cord post-delivery confirmed the presence of a single umbilical artery [15] (Fig. 2). All ultrasound examinations were carried out transabdominally by experienced operators using uniform high-resolution ultrasound equipment.

### Data collection

We conducted a review of computerized medical records for enrolled pregnant women to gather information on baseline maternal characteristics and associated perinatal complications, including maternal age, gravidity, parity, preconception body mass index (BMI), abnormal umbilical cord insertion (including velamentous cord insertion and marginal cord insertion), thrombophilia (a condition resulting from postnatal or genetically inherited factors causing blood abnormalities), meconium-stained amniotic fluid (characterized by green or yellowish appearance, indicating the presence of meconium), gestational diabetes mellitus (GDM, defined as fasting plasma glucose (FPG) ≥ 5.1 mmol/L, or 1-hour plasma glucose ≥ 10.0 mmol/L, or 2-hour plasma glucose ≥ 8.5 mmol/L at 24–28 weeks of gestation), and hypertensive disorders of pregnancy (HDP, including gestational hypertension defined as systolic blood pressure ≥ 140 mmHg and/or diastolic blood pressure ≥ 90 mmHg after 20 gestational weeks, and preeclampsia defined as hypertension after 20 gestational weeks with proteinuria, uteroplacental dysfunction, or organ damage; this group also includes women with conditions related to HDP, such as the HELLP syndrome) [16]. We also reviewed the coagulation profile in pregnant women at admission, including D-dimer, blood platelet count, prothrombin time (PT), activated partial thromboplastin time (APTT), and fibrinogen.

Perinatal outcomes included the rate of cesarean delivery, gestational age at delivery, preterm birth (defined as gestational age ≥ 28 weeks and < 37 weeks (196–258 days)), stillbirth, placental weight, fetal weight, fetal growth restriction (FGR, defined as birth weight less than the 10th percentile), macrosomia (defined as birth weight ≥ 4000 g), oligohydramnios (defined as amniotic fluid volume ≤ 300 ml, or the vertical depth of the maximum dark area of amniotic fluid under ultrasound was ≤ 2 cm or the amniotic fluid index was ≤ 5 cm in the third trimester of pregnancy), polyhydramnios (defined as amniotic fluid volume > 2000 ml), neonatal asphyxia (defined as low Apgar score or abnormal umbilical cord blood gas analysis or both), Apgar score at 1 min (based on heart rate, respiration, muscle tone, laryngeal reflex, and skin color within one minute after birth), Apgar score at 5 min (based on heart rate, respiration, muscle tone, laryngeal reflex, and skin color within five minutes after birth), intraamniotic infection (IAI, defined by standard clinical criteria, including maternal fever (≥ 38.0 °C) and at least one symptom such as maternal tachycardia > 100 bpm, fetal tachycardia > 160 bpm, uterine tenderness, maternal leukocytosis > 15,000 cells/mm^3^, and/or foul odor of the amniotic fluid) [16]. Additionally, we reviewed placentae and gestational membranes for histologic evidence of infection by postpartum pathology.

### Statistical analysis

Data were analyzed using SPSS 26.0 software (SPSS, Chicago, IL, USA). Normally distributed variables were presented as mean ± standard deviation and were analyzed by Student’s t test or one-way ANOVA. Nonnormally distributed variables were expressed as median and interquartile range and were analyzed by Mann-Whitney U test. Categorical variables were presented as number (%) and were analyzed by Pearson’s Chi-square test. Each variable was initially evaluated by univariate analysis including the chi-square test or one-way ANOVA. To identify risk factors for iSUA and UAT, multivariable logistic regression analysis was performed with a backward stepwise method for variables with *P* value < 0.2 in univariate analyses. Odds ratios (OR) and 95% confidence intervals (CI) were calculated. A power analysis with multiple regression model demonstrated that this study had a power of 95% in variable at significant level of 0.05. *P* value < 0.05 for multivariate regression analysis and perinatal outcomes was considered to be statistically significant.

## Results

A total of 46 pregnant women with umbilical artery thrombosis were identified during the study period, as well as 100 pregnant women with isolated single umbilical artery and 95 pregnant women with three umbilical vessels were selected as control group during the same period. The median gestational age at which umbilical artery thrombosis was detected in pregnancies was 29.71 weeks, with a quartile range of 22.96 to 34.29 weeks.

### Risk factors associated with UAT

As shown in Table 1, the proportions of abnormal cord insertion and thrombophilia were significantly higher in the UAT group compared to the three umbilical vessels group (*P* < 0.05). But there were no significant differences in maternal age, gravidity, parity, preconception BMI, GDM and HDP (*P* > 0.05). Table 2 showed the association between the coagulation profile and UAT. Blood platelet count, PT and fibrinogen in the UAT group were higher than those in three umbilical vessels group (*P* < 0.05). D-dimer and APTT were no different between the groups (*P* > 0.05).

**Table 1:** Comparison of maternal-related risk factors associated with UAT and iSUA

	Control (*n* = 95)	iSUA (*n* = 100)	UAT (*n* = 46)	*P*			
				NC vs. iSUA	NC vs. UAT	iSUA vs. UAT	
Maternal age (year)	30(27, 33)	31(27,33)	29(26,33)	0.433	0.513	0.358	
Gravidity	1(0,2)	1(0,2)	1(0,2)	0.608	0.434	0.230	
Parity	1(0,1)	1(0,1)	1(0,1)	0.473	0.706	0.843	
Preconception BMI (Kg/m^2^)	21.16(18.57, 23.40)	21.89(19.84, 23.38)	22.03(19.81, 26.02)	0.265	0.147	0.646	
Abnormal cord insertion	1 (1.1)	12 (12.0)	8 (17.4)	0.006	0.001	0.274	
Thrombophilia	1 (1.1)	1 (1.0)	3 (6.5)	0.953	0.040	0.032	
GDM	13 (14.6)	17 (17.0)	8 (17.4)	0.528	0.574	0.952	
Gestational hypothyroidism	2 (2.2)	3 (3.0)	1 (2.2)	0.691	0.981	0.768	
HDP	1 (1.1)	6 (6.0)	1 (2.2)	0.055	0.727	0.231	

Data were presented as median (IQR) or n (%)

GDM: Gestational diabetes mellitus; HDP: Hypertensive disorders of pregnancy

**Table 2:** Comparison of coagulation-related risk factors associated with UAT and iSUA

	Control (*n* = 95)	iSUA (*n* = 100)	UAT (*n* = 46)	*P*		
				NC vs. iSUA	NC vs. UAT	iSUA vs. UAT
D-dimer (ug/mL)	1.53 (1.21, 3.28)	1.66 (1.18, 4.30)	1.46 (0.89, 2.19)	0.427	0.645	0.271
Blood platelet count (*10^9^)	183.00(163.00, 209.00)	193.50(232.75, 164.25)	204.50(176.25,240.25)	0.151	0.027	0.236
PT (s)	10.60(11.10, 11.95)	11.95(11.00, 12.40)	11.60(11.00, 12.30)	0.001	0.028	0.445
APTT (s)	28.60(26.45, 20.95)	30.55(27.10, 32.70)	29.00(27.00, 32.50)	0.005	0.209	0.426
Fibrinogen(g/L)	4.35(3.98, 4.72)	4.40(3.99, 5.01)	4.54(4.03, 4.90)	0.138	0.035	0.865

Data were presented as median (IQR).

PT: Prothrombin time; APTT: Activated partial thromboplastin time

Univariate analysis identified preconception BMI, abnormal umbilical cord insertion, thrombophilia, blood platelet count, PT, and fibrinogen as significant predictors for subsequent multiple logistic regression analysis (*P* < 0.2) (Tables 1 and 2). The multiple logistic regression analysis showed that preconception BMI (OR [95%CI]: 1.212 [1.038–1.416], *P* = 0.015), abnormal umbilical cord insertion (OR [95%CI]: 16.695 [1.333-209.177], *P* = 0.029), thrombophilia (OR [95%CI]: 15.840 [1.112-223.699], *P* = 0.041) and PT (OR [95%CI]: 2.069 [1.091–3.924], *P* = 0.026) were statistically significant risk factors for UAT (Table 3).

**Table 3:** The multiple logistic analysis of risk factors associated with UAT

	NC (*n* = 95)	UAT (*n* = 46)	OR (95% CI)	
Preconception BMI (Kg/m^2^)	21.16(18.57, 23.40)	22.03(19.81, 26.02)	1.212(1.038–1.416)	0.015
Abnormal umbilical cord insertion	1 (1.1)	8 (17.4)	16.695(1.333-209.177)	0.029
Thrombophilia	1 (1.1)	3 (6.5)	15.840(1.112-223.699)	0.041
Blood platelet count (*10^9^)	183.00(163.00, 209.00)	204.50(176.25,240.25)	1.009(0.999–1.019)	0.072
PT (s)	10.60(11.10, 11.95)	11.60(11.00, 12.30)	2.069(1.091–3.924)	0.026
Fibrinogen(g/L)	4.35(3.98, 4.72)	4.54(4.03, 4.90)	1.526(0.970–2.399)	0.067

Data were presented as median (IQR) or n(%)

PT: Prothrombin time

Maternal characteristics and coagulation profile were compared between the iSUA and UAT populations in Tables 1 and 2. The results showed that the incidence of thrombophilia was higher in UAT group compared to iSUA group (*P* = 0.032). There were no significant differences in maternal age, gravidity, parity, preconception BMI, abnormal umbilical cord insertion, thrombophilia, GDM, HDP, D-dimer, platelet count, PT, APTT, and fibrinogen between iSUA and UAT groups (*P* > 0.05).

### Risk factors associated with iSUA

As shown in Table 1, compared to pregnancies with three umbilical vessels, the incidence of abnormal cord insertion was increased in iSUA group (*P* = 0.006), but there was no difference in maternal age, gravidity, parity, preconception BMI, thrombophilia, GDM and HDP between two groups (*P* > 0.05). As shown in Table 2, a comparative analysis of the coagulation profile was conducted between the iSUA group and three umbilical vessels group. PT and APTT of pregnant women at admission were higher in iSUA group than three umbilical vessels group (*P* < 0.05), while D-dimer, blood platelet count and fibrinogen were no difference between the groups (*P* > 0.05).

Univariate analysis identified abnormal cord insertion, HDP, blood platelet count, PT, APTT and fibrinogen as significant predictors for subsequent multiple logistic regression analysis (*P* < 0.2) (Tables 1 and 2). The multiple logistic regression analysis showed that abnormal umbilical cord insertion (OR [95%CI]: 15.043 [1.750-129.334], *P* = 0.014) were statistically significant risk factors for iSUA (Table 4).

**Table 4:** The multiple logistic analysis of risk factors associated with iSUA

	NC (*n* = 95)	iSUA (*n* = 100)	OR (95% CI)	*P*
Abnormal umbilical cord insertion	1 (1.1)	12 (12.0)	15.043(1.750-129.334)	0.014
HDP	1 (1.1)	6 (6.0)	8.724(0.956–79.636)	0.055
Blood platelet count (*10^9^)	183.00(163.00, 209.00)	193.50(232.75, 164.25)	1.005(0.999–1.012)	0.113
PT (s)	10.60(11.10, 11.95)	11.95(11.00, 12.40)	1.440(0.962–2.157)	0.076
APTT (s)	28.60(26.45, 20.95)	30.55(27.10, 32.70)	1.071(0.952–1.205)	0.255
Fibrinogen(g/L)	4.35(3.98, 4.72)	4.40(3.99, 5.01)	1.300(0.802–2.109)	0.287

Data were presented as median (IQR).

HDP: Hypertensive disorders of pregnancy; PT: Prothrombin time; APTT: Activated partial thromboplastin time

### Perinatal outcomes of pregnancies with UAT

Perinatal outcomes of pregnancies with UAT were reviewed and analyzed (Table 5). Compared with pregnancies with three umbilical vessels, pregnancies with UAT had a higher risk of cesarean delivery, preterm birth, oligohydramnios and IAI, and a lower weight of placenta and gestational age at delivery, and a greater likelihood of FGR as well as neonatal asphyxia in their newborns (*P* < 0.05). Compared with pregnancies with iSUA, pregnancies with UAT had a higher risk of cesarean delivery, preterm birth and IAI, and a lower weight of placenta and gestational age at delivery, and a greater likelihood of FGR and neonatal asphyxia in their newborns (*P* < 0.05). In addition, one stillbirth had occurred in the UAT group during the study period.

**Table 5:** Association of UAT and iSUA with perinatal outcomes

	NC (*n* = 95)	iSUA (*n* = 100)	UAT (*n* = 46)	*P*		
				NC vs. iSUA	NC vs. UAT	iSUA vs. UAT
Cesarean delivery	33 (37.1)	52 (43.7)	29 (63.0)	0.076	< 0.001	0.026
Gestational age at delivery (week)	39.39 ± 1.025	38.70 ± 1.656	37.07 ± 3.134	0.027	< 0.001	< 0.001
Preterm birth	1 (1.1)	10 (8.4)	20 (43.5)	0.210	< 0.001	< 0.001
Stillbirth	0 (0.0)	0 (0.0)	1 (2.2)	> 0.999	0.736	0.736
Weight of placenta (g)	512.58 ± 54.450	507.40 ± 69.584	469.78 ± 73.221	0.183	< 0.001	0.004
Fetal weight (g)	3342.67 ± 341.160	3068.66 ± 563.086	2756.20 ± 738.249	< 0.001	< 0.001	0.001
FGR	1 (1,1)	15 (12.6)	18 (39.1)	0.014	< 0.001	< 0.001
Macrosomia	2 (2.2)	4 (3.4)	2 (4.3)	0.712	0.460	0.654
Oligohydramnios	2 (2.2)	6 (5.0)	5 (10.9)	0.525	0.020	0.064
Polyhydramnios	0 (0.0)	1 (0.8)	1 (2.2)	> 0.999	0.060	0.058
Meconium-stained amniotic fluid	12 (13.5)	9 (7.6)	6 (3.6)	0.424	0.942	0.474
Neonatal asphyxia	0 (0.0)	7 (5.9)	10 (21.7)	0.058	< 0.001	0.001
Apgar score-1 min	10 (10, 10)	10 (10, 10)	10 (9, 10)	0.157	< 0.001	0.014
Apgar score-5 min	10 (10, 10)	10 (10, 10)	10 (10, 10)	0.552	0.012	0.040
IAI	0 (0.0)	20 (43.5)	17 (40.0)	< 0.001	< 0.001	< 0.001

Data were presented as mean ± SD, median (IQR) or n(%)

FGR: Fetal growth restriction; IAI: Intraamniotic infection

### Association of iSUA with perinatal outcomes

Perinatal outcomes of pregnancies with iSUA were reviewed and analyzed (Table 5). The risks of FGR and IAI were higher and the gestational age at delivery was lower in the iSUA group than in the three umbilical vessels group (*P* < 0.05), but there was no statistically significant difference in cesarean section, premature delivery, stillbirth, placental weight, macrosomia, oligohydramnios, meconium-stained amniotic fluid and neonatal asphyxia (*P* > 0.05).

## Discussion

This study demonstrated that preconception BMI, abnormal umbilical cord insertion, and thrombophilia were identified as risk factors for UAT, and an elongated prothrombin time (PT) was strongly associated with the occurrence of UAT. The risks of cesarean delivery, preterm birth, FGR, neonatal asphyxia, and IAI were higher in pregnancies with UAT than in pregnancies with three umbilical vessels or iSUA. Additionally, abnormal umbilical cord insertion was found to be associated with an elevated risk of iSUA, as well as an increased likelihood of FGR and IAI in pregnancies with iSUA compared to those with three umbilical cord vessels.

In this study, abnormal umbilical cord insertion, including velamentous and marginal cord insertions, was found to be a prevalent risk factor for both iSUA and UAT. The presence of abnormal placental umbilical cord insertion in pregnant women with UAT has been consistently reported by the majority of previous investigators. The abnormal insertion of the umbilical cord indeed has the potential to disrupt normal blood flow and increase the risk of thrombosis, both of which are considered potential causes of UAT [17]. Therefore, it is crucial to closely monitor pregnant women with abnormal umbilical cord insertion in order to promptly detect umbilical artery thrombosis.

Obesity stands as an independent risk factor for both arterial and venous thrombosis [18]. The mothers who are overweight prior to pregnancy were more prone to experiencing postpartum venous thrombosis [19]. The results of our study have also indicated that an elevated preconception BMI serves as a significant risk factor for UAT. Consequently, it suggests that pregnant women with an elevated preconception BMI face an increased susceptibility to UAT.

Our results indicated that thrombophilia was a risk factor for UAT. A case of inherited thrombophilia who developed UAT was reported [20]. During the pregnancy, a series of physiological alterations induce a hypercoagulable environment, which is deemed to be protective. However, the presence of inherited or acquired thrombophilia during gestation can disturb this intricate equilibrium, leading to an augmented inclination towards unwarranted thrombotic occurrences [21]. Therefore, it is imperative for expectant women with thrombophilia to exercise heightened vigilance regarding the potential risk of UAT.

An elongated PT may indicate abnormalities in certain factors within the coagulation system, which could increase the patient’s risk of thrombosis [22]. In this study, we observed a significant association between the prolongation of PT and the occurrence of UAT. Given the retrospective design of our study and the limitation of collecting PT measurements only at the time of admission, we were unable to establish a definitive causal relationship between PT prolongation and UAT. On the one hand, the elongation of PT in pregnant women may potentially indicate abnormalities in the coagulation system, which could increase the risk of UAT. On the other hand, it is also possible that the formation of UAT itself leads to PT prolongation. Nevertheless, the precise causal relationship between PT prolongation and UAT in pregnant women remains largely unexplored within the existing body of research. Further researches are needed to comprehensively investigate the potential causal relationship between PT and UAT, including examining the impact of PT values at different stages of pregnancy on the risk of UAT occurrence in pregnant women.

Diabetes and HDP are both conditions that can lead to endothelial injury, heightened inflammatory response, and platelet deposition on the vascular walls, thereby promoting thrombus formation. The presence of diabetes can also result in compromised blood flow within the small vessels and microcirculatory dysfunction. This, in turn, contributes to the development of thrombosis [23]. Thus, GDM and HDP may be independent risk factors for UAT [8, 24]. However, our study did not find a significant association between UAT and either HDP or GDM. This contradictory result may be attributed to differences in study design, study populations, and the severity of diabetes or hypertension. For instance, a considerable proportion of pregnant women with diabetes or HDP in this study exhibited well-controlled blood glucose or blood pressure level. Indeed, the precise timing of occurrence of UAT in relation to the onset of GDM and HDP in this study remains elusive due to the potential for ultrasound detection to indicate pre-existing thrombi.

Our study supported the existing literature regarding the association between iSUA and adverse pregnancy outcomes such as FGR and preterm birth [2, 25, 26]. Furthermore, we found that pregnancies with UAT had a higher risk of adverse perinatal outcomes, including cesarean delivery, FGR, preterm birth, neonatal asphyxia, stillbirth, and IAI compared to pregnancies with iSUA. These results indicate that UAT pregnancies are more susceptible to experiencing adverse perinatal outcomes than iSUA pregnancies, despite both populations presenting with a single umbilical artery on ultrasound examination and the absence of specific clinical symptoms or signs. Therefore, UAT poses a greater risk compared to iSUA, highlighting the importance of timely detection and early intervention in UAT pregnancies.

The umbilical blood vessels play a crucial role in transporting oxygen and essential substances to the fetus. The formation of a thrombus in the umbilical artery can result in reduced oxygen supply to the placental vessels, leading to a state of hypoxia [27]. This hypoxic condition can cause swelling of the intima, endothelial necrosis, and ultimately result in the occlusion of stem villi vessels and abnormal peripheral placental villi [27]. Many adverse pregnancy outcomes, such as FGR, preterm birth, neonatal asphyxia and stillbirth, can be attributed to abnormalities in placental trophoblast cells and placental dysfunction [28, 29]. This was why UAT was associated with adverse perinatal outcomes such as cesarean section, fetal growth restriction (FGR), preterm birth, neonatal asphyxia, and stillbirth [9, 10, 17, 27], which was consistent with our findings.

It is interesting to note that we observed a significant increase in the incidence of IAI and oligohydramnios in the UAT group, which has not been reported in other relevant studies. This may be that the impaired placental circulation caused by UAT could disrupt the normal exchange of nutrients and waste products between the mother and fetus, creating an environment conducive to infection or inadequate amniotic fluid production [30, 31]. Therefore, pregnant women who have UAT should be alert to the potential risks of oligohydramnios and IAI.

One of the strengths of this study is the inclusion of two control groups, allowing for comparisons of pregnant women with UAT to both the population with a normal three umbilical vessels and the population with iSUA. Additionally, our study was conducted as a multicenter study, which enhances the generalizability of the findings. However, it is important to acknowledge that our study was limited by its retrospective design. Therefore, to address this limitation, future research will focus on conducting prospective studies to further investigate the pregnancies with UAT.

## Conclusions

In conclusion, abnormal umbilical cord insertion serves as a common risk factor for both pregnancies with UAT and those with iSUA. Compared to pregnancies with iSUA or three-vessel umbilical cord, UAT pregnancies are associated with a higher risk of adverse perinatal outcomes.

## Data Availability

The datasets used and/or analysed during the current study are available from the corresponding author on reasonable request.

## List of Abbreviations

APTT: Activated partial thromboplastin time
BMI: Body mass index
CI: Confidence intervals
FGR: Fetal growth restriction
FPG: Fasting plasma glucose
GDM: Gestational diabetes mellitus
HDP: Hypertensive disorders of pregnancy
IAI: Intraamniotic infection
iSUA: Isolated single umbilical artery
OR: Odds ratios
PT: Prothrombin time
SUA: Single umbilical artery
UAT: Umbilical artery thrombosis
